# Electroanalytical Detection of Flavonoid Rutin Using a SPCE Modified with Manganese Oxide Recycled with Hydrothermal Treatment

**DOI:** 10.3390/nano16090537

**Published:** 2026-04-29

**Authors:** Gloria A. Cosco-Salguero, Carlos Castro-Rumiche, Johisner Penagos-Llanos, Rodrigo Segura, Edgar Nagles

**Affiliations:** 1 Facultad de Química e Ingeniería Química, Universidad Nacional Mayor de San Marcos, Lima 15081, Peru; gcoscos@unmsm.edu.pe (G.A.C.-S.); carlosupch@gmail.com (C.C.-R.); 2 Departamento de Química de los Materiales, Facultad de Química y Biología, Universidad de Santiago de Chile (USACH), Santiago 9170022, Chilerodrigo.segura@usach.cl (R.S.)

**Keywords:** MnO_2_ recycled, hydrothermal treatment, rutin, screen-printed electrode

## Abstract

This report presents a novel protocol for the recovery and utilization of recycled manganese oxide from used batteries through hydrothermal (HT) treatment. The recovered and treated material showed high activity towards rutin electro-oxidation (RT) on a screen-printed carbon electrode (SPCE) modified with recovered and treated MnO_2_ within a hydrothermal reactor. Material characterization using scanning electron microscopy (SEM) and density distribution and particle size analysis revealed a more homogeneous and less dispersed particle size compared to the untreated material. This treatment increased the electroactive activity of the anodic current for RT by more than 70% compared to the SPCE without MnO_2_ treated with HT. The electroanalytical application of this new electrode enabled the detection of RT, with a detection limit of 0.03 µmol/L, and its application in natural samples such as coffee and flavored beverages.

## 1. Introduction

Rutin is one of the most studied flavonoids due to its significant health benefits. Its biological and pharmacological properties have been reported for decades [1]. Several reviews highlight the capabilities of this flavonoid, primarily as a cancer treatment [2,3], for its antidiabetic effects [4], for disease prevention [5], as a promising nutraceutical agent [6,7], and as a potent antioxidant [8]. All these recent reports about the health benefits of rutin justify its study and detection in all kinds of natural foods. One of the techniques used to detect rutin is HPLC where reported detection limits for rutin below 1.0 µmol/L with a UV–VIS detector [9], offering high sensitivity but also high operating and instrumental costs.

Electroanalytical techniques have proven to be equally sensitive, selective, and have a lower operating cost. In this context, various materials have been reported for the electroanalytical detection of rutin, some carbon derivatives such as carbon nanotubes [10], carbon nanotubes with ionic liquid [11], graphene with ionic liquid [12], metal cation complexes such as ferrocene [13], gold nanoparticles [14,15], platinum [16], metal cation oxides such as neodymium [17] and polymers such poly(acridine orange), [18], polypyrrole [19] and as poly 3,4-ethylenedioxythiophene [20]. These reports indicate detection limits between 0.005 and 0.2 µmol/L where the most commonly used and most sensitive materials were combined with ionic liquids. On the other hand, the few reports using a manganese oxide-modified electrode to detect rutin combine graphene and ionic liquid [21] and fungus-derived biomass [22] with detection limits below 0.02 µmol/L. These reports provide evidence of the conductive and catalytic capabilities of MnO_2_. It is also low-cost, abundant in nature, low-toxicity, and has a wide potential range [22]. Therefore, the use of MnO_2_ in electrode modification has increased in recent years as a capacitor material [23] and for the detection of tartrazine [24], amaranth [25], peroxide [26], and the antipsychotic drug olanzapine [27].

The application of hydrothermal treatment for obtained MnO2 has been developed primarily to improve its properties as a capacitor in lithium batteries where the main precursor is KMnO_4_, and where the electrochemical properties of the synthesized MnO_2_ differ depending on the morphology of the crystals formed [28]. Furthermore, the use of MnO_2_ recycled from batteries was first reported in 2026 where was combined with carbon powder for the development of a modified electrode to detect paracetamol [29]. In this new report, the hydrothermal treatment was developed with the objective of obtaining a more homogeneous and smaller particle size material compared to the MnO_2_ obtained from the recycled battery without hydrothermal treatment, and that improves the sensitivity and selectivity in the detection of rutin in real samples and that it is equally sensitive compared to other materials conventionally used for routine detection such as carbon nanotubes, graphene, and ionic liquids.

## 2. Materials and Methods

### 2.1. Reagents and Instruments

A Thermo Scientific Barnstead MicroPure-ST system (USA) was used to obtain ultrapure water to prepare all the solutions. MnO_2_ was extracted from a used D-type battery. Reagents such as rutin (RT), morin (MO), quercetin (QC), K_4/_K_3_Fe(CN)_6_, chitosan, KCl, NaH_2_PO_4_ and H_3_PO_4_ were obtained from Sigma Aldrich. (Darmstadt, Germany) A screen-printed electrode DRP 104 obtained from Dropsens Metrohm (Herisau, Switzerland) was used as the integrated electrode system with carbon as the working electrode and auxiliary electrode a Ag as electrode reference (RE). Cyclic and square-wave voltammetry (CV, SWV) were performed using a Dropsens µ-Stat 400i potentiostat (Metrohm). SEM and energy-dispersive X-ray spectroscopy (EDS) images were acquired using a high-resolution scanning electron microscope (Inspect-F50; Thermo Fisher Scientific, FEI, Eindhoven, The Netherlands). The particle size was determined by applying the dynamic light scattering method using a Winner802 particle size analyzer. A PTFE-lined hydrothermal synthesis autoclave reactor was used (Model: KH-25 from Haocheng Instruments, Kobe-shi, Japan). A PTFE-lined hydrothermal synthesis autoclave reactor was used (Model: KH-25 from Haocheng Instruments) (Shenzhen, China).

### 2.2. Measurements

CV and SWV measurements were performed using a 10 mL glass cell containing 8.0 mL of ultrapure aqueous sodium chloride, 0.5 mL of phosphate-buffered solution (PBS), and 0.25 mL of RT 10.0 mmol/L for CV and 20.0 µL of RT 1.0 mmol/L for SWV. The K_4_/_3_Fe(CN)_6_ solution used in the activity and active area studies was 10.0 mmol/L in 0.10 mmol/L KCl. CV was performed at a scan rate of 0.1 V/s with a sweep range from 0.0 to 1.2 V. The initial parameters for SWV are given in the optimal parameters section.

### 2.3. Sample Treatment

Samples of coffee and tea were dried to between 2.0 and 3.0 g, then 100 mL of hot water was added to extract flavonoids. A total of 0.1 mL of the sample extract was added to the cell for analysis. All samples were obtained from a supermarket in the city of Lima. No pretreatment was performed.

### 2.4. Electrode Preparation

**Scheme 1:**
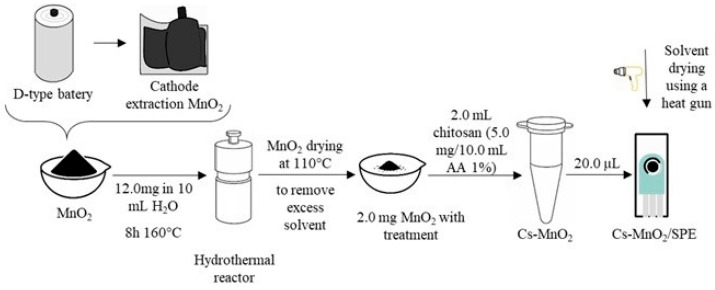
Preparation electrode.

SPCE was treated by CV with two cycles at a sweep speed of 0.10 V/s between 0.0 and 1.0 V (vs. Ag RE) with a pH 7.0 phosphate-buffered solution before each modification. This pretreatment proved effective in obtaining stable measurements. The material optimization and electrode preparation processes are summarized in Scheme 1. The indicated quantities of MnO_2_, temperature, and time were optimal toward the maximum anodic peak currents of RT. The chitosan is prepared as previously reported [24]. A total of 5.0 mg of low molecular weight chitosan are dissolved in 10.0 mL of acetic acid (AA) 1.0%

## 3. Results and Discussion

### 3.1. Measurable Changes in the Electrodes Modified with MnO_2_ Recycle Before and After Hydrothermal Treatment

Hydrothermal treatment with liquid phase using water ultrapure in a hydrothermal autoclave offers great advantages when the aim is to reduce and control the particle size of different substances dispersed in a liquid phase [30] where nanoparticles can be obtained with long treatment times, but with the disadvantage that the process consumes high amounts of energy. In this case, the material was subjected to a few hours of treatment at low temperatures. This process did not produce a nanomaterial, but it was sufficient to achieve high activity towards RT electro-oxidation. The material obtained was used to modify a SPCE as shown in Scheme 1 and applied in the detection of RT in aromatic beverages.

**Figure 1:**
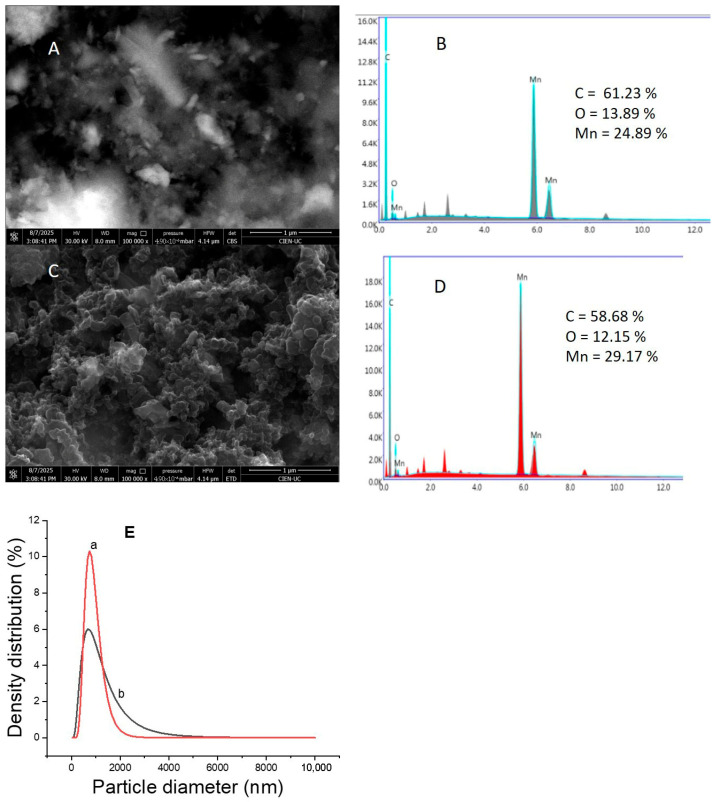
SEM images of the untreated material (**A**) and treated material (**C**), EDS images of the untreated material (**B**) and treated material. (**D**,**E**) Density distribution and particle size for the untreated material (curve b) and treated material (curve a).

The authors characterized the material extracted from battery D before and after hydrothermal treatment by SEM, EDS and particle size analysis. The results are shown in Figure 1. SEM images of the material before and after treatment are shown in Figure 1A,C. Before treatment, the particles were irregularly shaped like flakes and varied in size (Figure 1A), while after treatment, the material appeared more uniform in size and shape, with circular particles instead of flakes (Figure 1C). This result demonstrates the control that this treatment can exert over the shape and size of the materials. Furthermore, the EDS image of the untreated material (Figure 1B) shows an oxygen content of 13.89% and a manganese content of 24.89%. With these values, the oxygen-to-manganese mass ratio was 0.56. This value is close to the theoretical O/Mn mass ratio for MnO_2_, which is 0.58. For the treated material (Figure 1D), the O/Mn mass ratios were 12.15% for O and 24.89% for Mn, with an O/Mn mass ratio of 0.42. This value is much lower than that observed for the untreated material and close to the theoretical value of 0.43 for Mn_2_O_3_. This result indicates that the initial material underwent changes, and a change in the oxidation state of the manganese in the recycled battery occurred, shifting from Mn^2+^ to Mn^3+^. This type of phenomenon is normal, as previous studies on the hydrothermal synthesis of MnO_2_ materials have shown the formation of other manganese materials with different oxidation states, such as Mn^4+^ [31].

To determine the changes in particle size and shape observed in the SEM images before and after treatment, particle size was measured using a Winner802 particle size analyzer. The results are shown in Figure 1E. The treated and untreated material was dispersed in water and measured by dynamic light scattering. The results show that the untreated material (curve b in Figure 1E) has particles of varying sizes, between 1000 and 10,000 nm, with an average size of 1390 nm. On the other hand, when the material underwent hydrothermal treatment, a smaller particle size was observed (curve a; Figure 1E), with an average size of 930 nm. The peak shapes show a taller and thinner peak for the particle size of the treated material, indicating smaller and more uniform particles. These results indicate that the hydrothermal treatment helped improve the homogeneous and reduced particle size distribution under these conditions. This particle size may explain the effect on the electrochemical activity of RT on a SPCE modified with this treated material.

### 3.2. Characterization of the SPCE and Cs-MnO_2_/SPCE by CV

**Figure 2:**
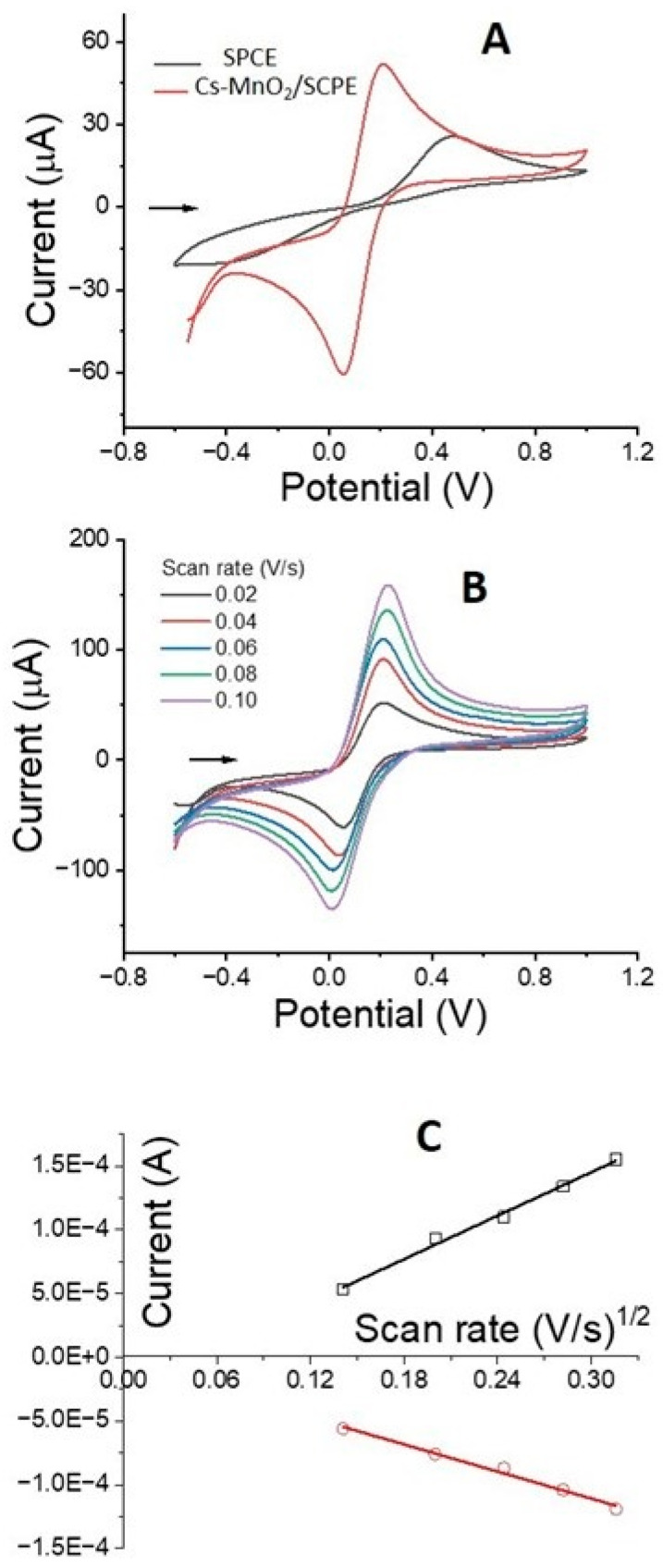
(**A**) Cyclic voltammograms of K_4/3_Fe(CN)_6_ 0.05 mmol/L with SPCE and Cs-MnO_2_/SPCE at a scan rate of 0.04 V/s; (**B**) Cyclic voltammograms of K_4/3_Fe(CN)_6_ with Cs-MnO_2_/SPCE at scan rates between 0.02 and 0.10 V/s and (**C**) plots of Ip(µA) vs. scan rate^1/2^. Experimental conditions: K_4/3_Fe(CN)_6_ (5.0 mmol/L) with 0.10 mol/L KCl using Ag as reference electrode.

With the aim of identifying the benefits to the electroactive and active surface properties of a SPCE due to the presence of the treated MnO_2_ material, several CV studies were conducted using K_4/3_Fe(CN)_6_ 5.0 mmol/L as the test redox system. The results are shown in Figure 2. First, the study examined how the presence of the material improves the charge transfer rate and decreases the energy required for the oxidation-reduction in the test redox system.

These results are shown in Figure 2A, where the significant electroactive effect on the carbon electrode material modified with the material is clearly observed. A significant increase in anodic and cathodic currents was observed; furthermore, the anodic and peak cathodic currents values were almost equal, presenting an ΔI close to 1.0, indicating a nearly identical kinetic system for both processes. On the other hand, the anodic and cathodic peak potential values are lower, with the ΔV decreasing from 0.78 V with SPCE alone (Figure 2A, black curve) to 0.15 V with SPCE with the treated material Cs-MnO_2_/SPCE (Figure 2A, red curve). These results are evidence that the test redox system becomes less irreversible in the presence of the MnO_2_-treated material. The potential and current changes in Figure 2A, with the SPCE and Cs-MnO_2_/SPCEs, are summarized in Table 1. To verify any changes on the surface of the modified electrode, a study was conducted as a function of the scan rate between 0.02 and 0.10 V/s per CV for SPCE alone and SPCE modified with MnO_2_. The results are shown in Figure 2B,C for SPCE modified with MnO_2_. The results clearly show that the anodic and cathodic peak currents increase proportionally with increasing scan rate (Figure 2B), but do not exhibit a linear trend because the background current also increases. However, the linear relationship between the anodic and cathodic peak currents and the square root of the scan rate (Figure 2C) shows a linear trend with an R^2^ close to 1.0, indicating a diffusion-controlled process. After all, the fact that the process is diffusion-controlled allows us to calculate the active area of the modified electrode using the Randles–Sevcik equation, where the concentration of the test redox system and its diffusion coefficient are known. The active area calculated using anodic and cathodic slope values of 5.67 × 10^−4^ and 3.53 × 10^−4^ I_p_(A)/(V/s)^1/2^ (Figure 2C) was 0.15 and 0.09 cm^2^, respectively, for an average active area of 0.12 cm^2^ for the MnO_2_-treated SPCE modified. Furthermore, the active area for SPCE alone was 0.066 cm^2^. This result represents an almost 100% increase in the active area. This increase may explain the observed effect of higher currents and improved electrostatic interaction between the redox system and the treated MnO_2_. In relation to the calculated active area, with SPCE modified with MnO_2_ without hydrothermal treatment, an active area value of less than 0.080 cm^2^ has been reported [24] and with carbon paste-modified MnO_2_, an area slightly greater than 0.16 cm^2^ has been reported [29].

### 3.3. Study of Rutin Activity on SPCE and Cs-MnO_2_/SPCE by CV

**Figure 3:**
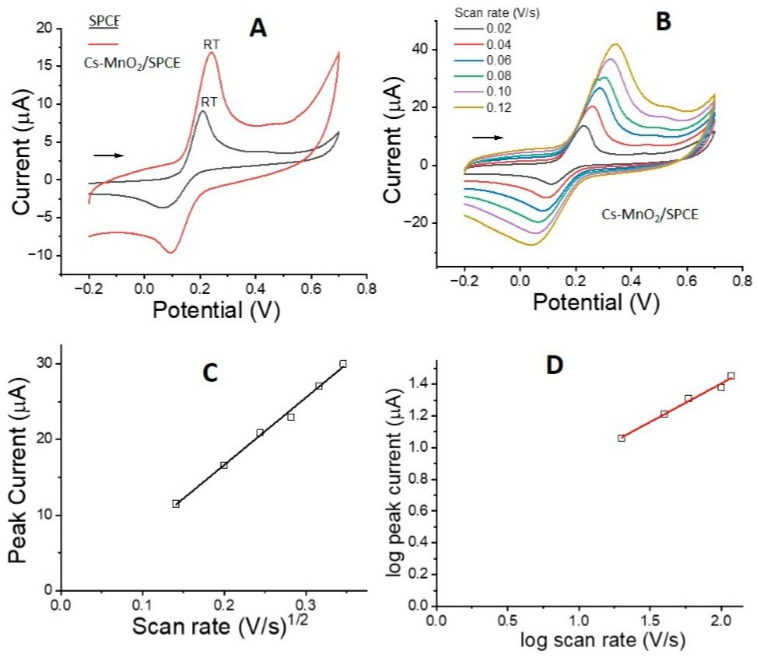
(**A**) Cyclic voltammograms of the RT 0.25 mmol/L with SPCE, and Cs-MnO_2_/SPCE at a scan rate of 0.02 V/s, (**B**) cyclic voltammograms of the RT with Cs-MnO_2_/SPCE at scan rates of 0.02–0.12 V/s, and plots of (**C**) I_p_(µA) vs. the scan rate^1/2^ (V/s) and (**D**) log I_p_(µA) vs. the log scan rate (mV/s). Experimental conditions: RT (0.25 mmol/L) and a pH of 3.0 in PBS, using Ag as reference electrode.

Rutin is a pentahydroxyflavone that is normally reversibly oxidized at potential values between 0.2 and 0.8 V on carbon electrodes. In this case, the electroactivity of RT 0.25 mmol/L was evaluated on SCPE and SPCE modified with MnO_2_-treated material by cyclic voltammography. The voltammograms are shown in Figure 3A, and the voltammograms for each scan rate between 0.02 and 0.12 V/s, along with plots for I_p_ (µA) vs. square root of scan rate and log I_p_ (µA) vs. log scan rate, are shown in Figure 3B–D. The electrodes were immersed in the same RT 0.25 mmol/L solution with phosphate buffer (PBS) solution at pH 3.0, and the potential was scanned between −0.2 and 0.70 V/s. At 0.02 V/s, a redox reaction for RT was observed between 0.21 and 0.24 V for SPCE and CsMnO_2_/SPCE, respectively, but a considerable increase in anodic current for RT with CsMnO_2_/SPCE was observed, increasing from 7.26 to 12.76 µA. This represents an increase of more than 70% in the anodic current of RT. On the other hand, the potential values showed almost no change. This result indicates that the rate of charge transfer for RT is greater with the modified electrode, possibly due to a greater electrostatic interaction and a higher concentration of RT on the electrode surface, possibly due to the increased active surface area. These results show greater activity towards the oxidation of RT with the electrode modified with the MnO_2_ material. This is positive as it allows the development of a methodology for detecting RT with a lower detection limit. Regarding the observed potential value, the potential observed with this modified electrode was lower compared to other modified electrodes such as graphene and gold nanoparticles [14], platinum nanoparticles–graphene [16], gold nanoparticles/ethylenediamine/carbon nanotube [15], and graphene quantum dots (GQDs) and poly(3,4-ethylenedioxythiophene) (PEDOT) [20] and graphene–carbon nanotubes [19]. This indicates that this modified electrode requires less energy for RT oxidation. But with modified electrodes containing MnO_2_, the potential value is close to that reported in this new study, less than 0.3 V [22]. Studies for RT with the electrode modified with recycled manganese oxide without hydrothermal treatment showed no activity towards RT electro-oxidation. Therefore, hydrothermal treatment was important to improve the activity of the modified electrode.

### 3.4. Effect of pH

**Figure 4:**
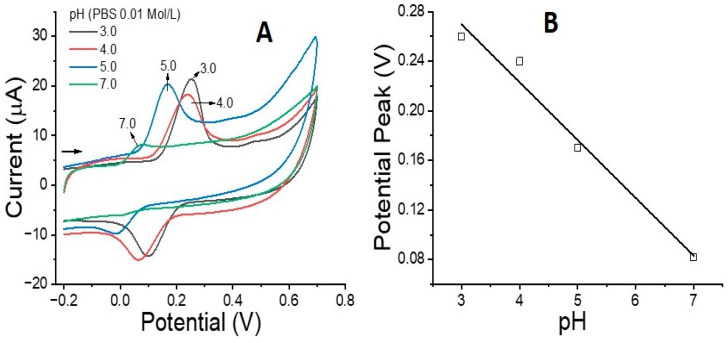
(**A**) Cyclic voltammograms of RT (0.25 mmol/L) with Cs-MnO_2_/SPCE at different pH values (**B**) Plot of the pH vs. the anodic peak potential E_p_(V) for RT (0.25 mmol/L). Experimental conditions: scan rate of 0.04 V/s, using Ag as reference electrode.

The effect of pH on the peak anodic current for RT (0.25 mmol/L) with Cs-MnO_2_/SCPE was studied between pH 3.0 and 7.0 with PBS and by CV at 0.04 V/s. The voltammograms are shown in Figure 4A, and the plot of pH vs. E_p_ (V) is shown in Figure 4B. The results show the normal trend observed when protons are being oxidized in the redox process, where the potential value decreases as the pH value increases. These results indicate that RT exhibits Nernstian behavior and that H^+^ is oxidized in the structure. The fitted relationship for the RT curve was:

Ep(V) = −0.0464 pH + 0.4029

The slope of the curve is close to the value for the Nernst constant, indicating that the redox reactions have the same H^+^/e^−^ ratio that is normally reported as 2H^+^/2e^−^. These results were similar to those obtained with other, more complex electrodes [32].

### 3.5. Optimization of Parameters and the Calibration Curve

**Figure 5:**
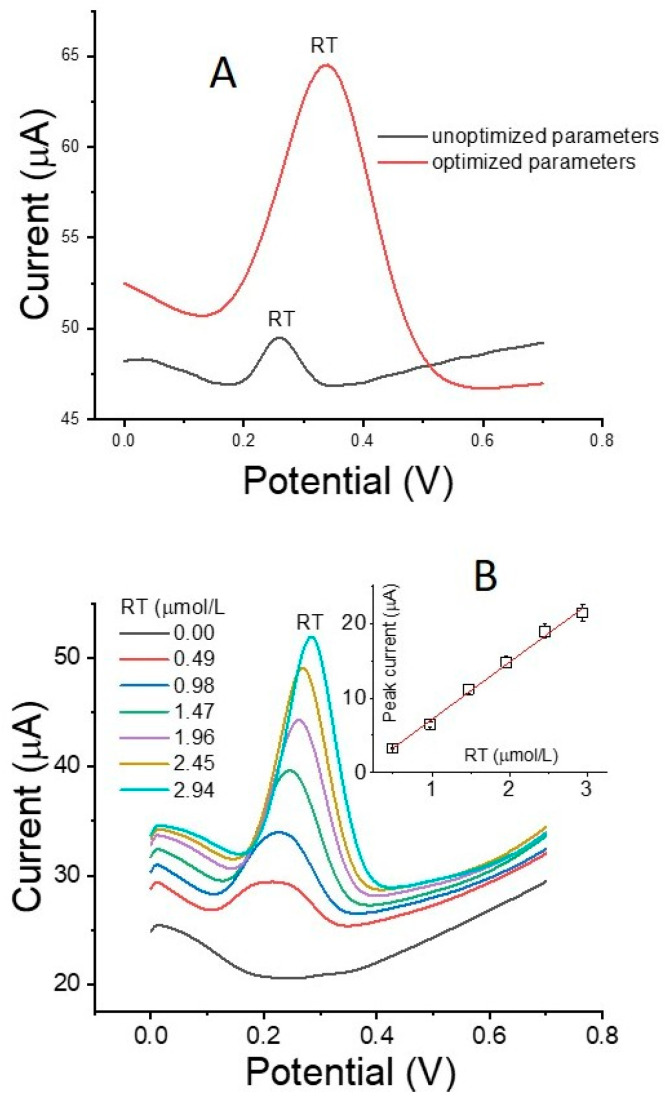
(**A**) Square-wave voltammograms of SWV with optimized parameters (red curve) and unoptimized parameters (black curve) for RT (5.5 µmol/L), and (**B**) square-wave voltammograms and the corresponding calibration curve (inset) for RT. Concentrations ranging from 0.5 to 3.0 µmol/L were determined using the Cs-MnO_2_/SPCE. Experimental conditions: pH, 3.0; E_ACC,_ +0.1 V for 30.0 s; frequency, 5.0 Hz; E_Step_, 0.005 V; and E_amplitude_, 0.1 V, using Ag as reference electrode.

With the aim of increasing RT oxidation activity and improving sensitivity, SWV was used to simultaneously study the variables that most affect the anodic current, such as step potential (E_Step_), potential amplitude (E_Amplitude_), and frequency (F) for the sweep stage, and deposition time (t_ACC_) and deposition potential (E_ACC_) for the accumulation stage. The possible combinations of variables and the number of experiments, along with the corresponding current values, are summarized in Table 2. The results in Table 2 clearly show that the parameters that most affect the anodic current were those that control the potential scan, especially step potential and frequency. Specifically, the peak anodic currents for RT were higher at lower frequencies and step potentials between 5.0 Hz and 0.005 V. A comparison of the peak anodic currents for the optimized parameters (red curve) and the non-optimized parameters (black curve) is shown in Figure 5A. The increase in peak anodic current for RT was greater than 200%, with a shift in the peak potential toward more positive values. Therefore, 5.0 Hz, a step potential of 0.005 V, and a potential amplitude of 0.10 V with a build-up potential of +0.1 V for 30 s were considered optimal and were chosen for developing the calibration curves.

The calibration curves measured for RT are shown in Figure 5B, where the plots of Ip (µA) vs. concentration (µmol/L) show an R^2^ of 0.991 for RT. The detection limits calculated from the slope of the calibration curve (3σ/slope) were 0.030 µmol/L, with sensitivities of 7.685 µA/µmol^−1^. The sensitivity results were almost identical to those of the other modified electrodes, which are summarized in Table 3.

### 3.6. Stability, Reproducibility, and Selectivity

**Figure 6:**
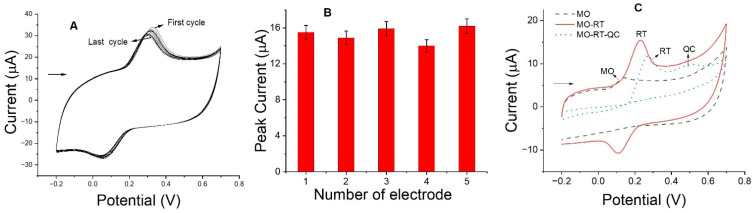
(**A**) Cyclic voltammograms (30 times) of 0.25 µmol/L RT. (**B**) Anodic peak currents RT with five different electrodes and (**C**) Cyclic voltammograms for MO (dash line), MO-RT (solid line) and MO-RT-QC (dotted line). The experimental conditions were pH 3.0 PBS, scan rate 0.10 V/s and MO, RT and QC 0.25 mmol/L.

The stability, reproducibility, and selectivity of the new modified electrode for RT detection were evaluated through three independent studies. The results are shown in Figure 6. First, to verify the stability of the modified electrode surface, thirty consecutive cyclic voltammograms were developed for RT (0.25 mmol/L) in pH 3.0 PBS. The results showed (Figure 6A) a decrease in the peak anodic current for RT of almost 5.0 µA. This indicates a variation of almost 0.16 µA per cycle. Therefore, the modified electrode shows high stability and can be used in more than ten consecutive measurements without significant loss of activity. Second, reproducibility was evaluated with five electrodes modified in the same way to assess the variation in the anodic current for RT (0.25 mmol/L). The results are shown in Figure 6B. A small variation in current was clearly observed for each electrode, ranging from 14.6 µA to 16.1 µA, with an average value of 15.5 µA. The relative standard deviation was 0.52, and the percentage variation coefficient was 3.2%. These results indicate a variation of less than 10.0% between each observed current value for each modified electrode. Finally, to verify the selectivity of the modified electrode for RT detection, its activity was evaluated against other substances, such as dopamine (DA), uric acid (UA), ascorbic acid (AA), hydroquinone (HQ), catechol (CT), morin (MO), and quercetin (QC). The results showed that the only substances exhibiting activity that could potentially interfere with RT detection were MO and QC. Therefore, the cyclic voltammograms of RT in the presence of MO and QC are shown in Figure 6C. The results showed that MO (dash line) is electro-oxidized at lower potential values than RT, but the signal is very small compared to the RT signal. When RT is added to the same cell, a considerable increase is observed due to the electro-oxidation of RT, which displaces the MO signal, possibly due to a competitive effect that is more favorable to RT (solid line). When QC is added to the same cell (dotted line), a small, broad signal is observed at potential values greater than 0.4 V, which interferes with the RT signal. The concentrations of MO and QC were 0.25 mmol/L. This result is important because MO and QC are other flavonoids with chemical structures very similar to that of RT. In addition, MO and RT are present in the real samples analyzed. In this case, the electrode proved to be stable, reproducible, and selective for the detection of RT.

### 3.7. Analytical Application in Real Samples

**Figure 7:**
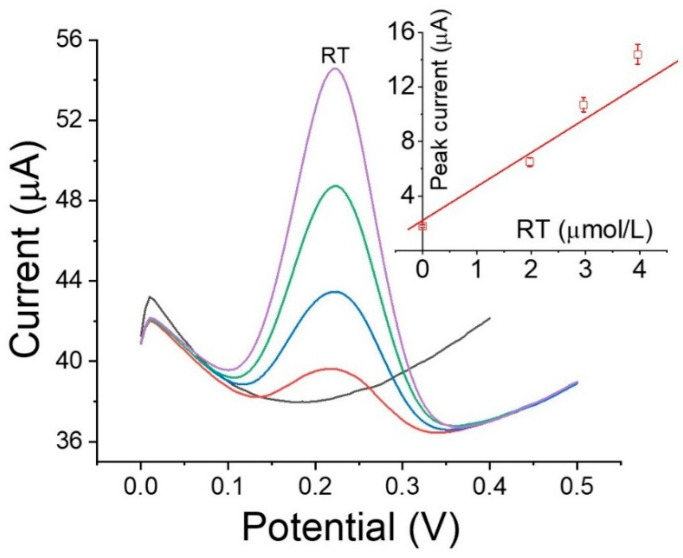
Square-wave voltammograms for the drinking water sample spiked with RT 1.0 µmol/L obtained using the Cs-MnO_2_/SPCE, along with the calibration plot (inset). The conditions are consistent with those described in Figure 5.

The actual samples were applied to study the accuracy of the new method using the modified electrode with drinking water samples doped with known amounts of RT. A total of 1.0 µmol/L was added to the samples. The voltammograms and calibration curve of one of the samples are shown in Figure 7. An RT concentration of 0.98 ± 0.01 µmol/L was calculated with a relative error of −2.0% and a recovery percentage of 98.0%. The results showed acceptable accuracy. The actual samples studied were coffee and tea extracts obtained after treating 2.19 g of coffee and 3.63 g of tea with 50.0 mL of ultrapure hot water and filtering. The analyses were performed in triplicate using the standard samples. The calculated values for coffee and tea were 79.7 ± 0.20 µmol/L and 55.0 ± 0.10 µmol/L for coffee and tea, respectively. These values indicate a total flavonoid content of 0.048 g/L and 0.033 g/L in coffee and tea, respectively. These values were very close to those reported for coffee using SPCE modified with other materials such as walled carbon nanotubes [33] and studies of the presence of these flavonoids based on the origin and treatment of the coffee bean [34].

## 4. Conclusions

This research allowed adding value to a recycled material for the development of a modified electrode applied to the detection of RT in food with a detection limit below 0.1 µmol/L with high selectivity and reproducibility. The results show the positive effect that hydrothermal treatment can have on improving the activity of cation oxides such as manganese, because it allows obtaining more homogeneous and smaller particles, improving their activity in the development of modified electrodes that can exhibit activity very similar to other modified electrodes that are more complex to manufacture.

## Figures and Tables

**Table 1:** Anodic and cathodic peak and potential currents for K_4_/K_3_Fe(CN)_6_.

Electrode	Currents Ip (µA)		Potential Ep (V)		% Current Increase		ΔIp
	Ipa	Ipc	Ea	Ec			
CPE	94.8	−93	0.31	0.092	-	-	1.03
Nd_OX_/CPE	130.4	−123	0.35	0.066	30	25	1.05
_HT_Nd_OX_/CPE	289.0	−256	0.32	0.068	300	270	1.12

**Table 2:** Variables that most affect the anodic current for the RT using Cs-MnO_2_/SPCE.

Experiments	F (Hz)	E_Step_ (V)	E_Amplitude_ (V)	t_ACCC_ (s)	E_ACC_ (V)	I_p_ (µA)
* 1	20	0.01	0.05	10	0	2.61
2	5	0.01	0.05	10	0	5.71
3	2	0.01	0.05	10	0	3.65
4	5	0.005	0.05	10	0	6.65
5	5	0.005	0.02	10	0	6.02
6	5	0.005	0.07	10	0	9.85
7	5	0.005	0.09	10	0	11.33
8	5	0.005	0.10	10	0	11.72
9	5	0.005	0.10	20	0	12.76
10	5	0.005	0.10	30	0	13.56
11	5	0.005	0.10	30	−0.1	13.50
* 12	5	0.005	0.10	30	+0.1	15.63
13	5	0.005	0.10	30	+0.2	13.40

* voltammograms shown in Figure 5A.

**Table 3:** Modified electrodes for the RT.

Electrode	Technique	Application	LoD (µmol/L)		Ref.
			DA	PCM	
MWCNT/GCE	DPV	Human serum	0.800	0.600	[19]
MWCNT-GO/GCE	DPV	Commercially samples	0.022	0.047	[20]
MWCNT-CoNP/GCE	DPV	pharmaceuticals	0.001	0.015	[30]
Cu-MOFs/MWCNT-Au@Ag/GCE			0.082	0.230	[21]
Gr/CNF-AgNPS	DPV	human urine, blood serum	0.034	0.019	[22]
rGO-PVP/GCE	SWV	artificial urine, pharmaceutical syrup, energy beverage	0.810	0.160	[23]
CoFe_2_O_4_/GP MnFe_2_O_4_/GP	DPV	urine	0.250 0.400	0.350 0.300	[24]
_HT_Nd_OX_/CPE	SWV	artificial urine, human urine pharmaceutical syrup	0.060	0.020	This work

## Data Availability

Data are contained within the article.

## References

[1] Negahdari R., Bohlouli S., Sharifi S., Dizaj S.M., Saadat Y.R., Khezri K., Jafari S., Ahmadian E., Jahandizi N.G., Raeesi S. (2021). Therapeutic benefits of rutin and its nanoformulations. Phytother. Res..

[2] Imani A., Maleki N., Bohlouli S., Kouhsoltani M., Sharifi S., Dizaj S.M. (2021). Molecular mechanisms of anticancer effect of rutin. Phytother. Res..

[3] Farha A.K., Gan R.Y., Li H.B., Wu D.T., Atanasov A.G., Gul K., Zhang J.R., Yang Q.Q., Corke H. (2022). The anticancer potential of the dietary polyphenol rutin: Current status, challenges, and perspectives. Crit. Rev. Food Sci. Nutr..

[4] Ghorbani A. (2017). Mechanisms of antidiabetic effects of flavonoid rutin. Biomed. Pharmacother..

[5] Patel K., Patel D.K., Watson R.R., Preedy V.R. (2019). Chapter 26—The Beneficial Role of Rutin, A Naturally Occurring Flavonoid in Health Promotion and Disease Prevention: A Systematic Review and Update. Bioactive Food as Dietary Interventions for Arthritis and Related Inflammatory Diseases.

[6] Prasad R., Prasad S.B. (2019). A review on the chemistry and biological properties of Rutin, a promising nutraceutical agent. Asian J. Pharm. Pharmac..

[7] Ravindra S., Kumar J.S., Badoni S.R., Kumar S.D. (2021). Health benefits and limitations of rutin—A natural flavonoid with high nutraceutical value. Phytochem. Lett..

[8] Enogieru A.B., Haylett W., Hiss D.C., Bardien S., Ekpo O.E. (2018). Rutin as a Potent Antioxidant: Implications for Neurodegenerative Disorders. Oxid. Med. Cell. Longev..

[9] Jan S., Ahmad J., Dar M.M., Wani A.A., Tahir I., Kamili A.N. (2022). Development and validation of a reverse phase HPLC–DAD method for separation, detection & quantification of rutin and quercetin in buckwheat (*Fagopyrum* spp.). J. Food Sci. Technol..

[10] Zeng B., Wei S., Xiao F., Zhao F. (2006). Voltammetric behavior and determination of rutin at a single-walled carbon nanotubes modified gold electrode. Sens. Actuat. B Chem..

[11] Liu X., Li L., Zhao X., Lu X. (2010). Electrochemical behavior of rutin on a multi-walled carbon nanotube and ionic liquid composite film modified electrode. Colloids Surf. B Biointerfaces.

[12] Gao F., Qi X., Cai X., Wang Q., Gao F., Sun W. (2012). Electrochemically reduced graphene modified carbon ionic liquid electrode for the sensitive sensing of rutin. Thin. Solid Films.

[13] Liu M., Deng J., Chen Q., Huang Y., Wang L., Zhao Y., Zhang Y., Li H., Yao S. (2013). Sensitive detection of rutin with novel ferrocene benzyne derivative modified electrodes. Biosens. Bioelectron..

[14] Niu X., Wen Z., Li X., Zhao W., Li X., Huang Y., Li Q., Li G., Sun W. (2018). Fabrication of graphene and gold nanoparticle modified acupuncture needle electrode and its application in rutin analysis. Sens. Actuat. B Chem..

[15] Yang S., Qu L., Li G., Yang R., Liu C. (2010). Gold nanoparticles/ethylenediamine/carbon nanotube modified glassy carbon electrode as the voltammetric sensor for selective determination of rutin in the presence of ascorbic acid. J. Electroanal. Chem..

[16] Yu S.H., Zhao G.C. (2012). Preparation of Platinum Nanoparticles-Graphene Modified Electrode and Selective Determination of Rutin. Int. J. Electrochem..

[17] Nagles E., Calderón J.A., García-Beltrán O. (2017). Development of an electrochemical sensor to detect dopamine and ascorbic acid based on neodymium (III) oxide and chitosan. Electroanalysis.

[18] Sun W., Wang Y., Gong S., Cheng Y., Shi F., Sun Z. (2013). Application of poly(acridine orange) and graphene modified carbon/ionic liquid paste electrode for the sensitive electrochemical detection of rutin. Electrochim. Acta.

[19] Yang L., Yang J., Xu B., Zhao F., Zeng B. (2016). Facile preparation of molecularly imprinted polypyrrole-graphene-multiwalled carbon nanotubes composite film modified electrode for rutin sensing. Talanta.

[20] Meng R., Li Q., Zhang S., Tang J., Ma C., Jin R. (2019). GQDs/PEDOT Bilayer Films Modified Electrode as a Novel Electrochemical Sensing Platform for Rutin Detection. Int. J. Electrochem. Sci..

[21] Sun W., Wang X., Zhu H., Sun X., Shi F., Li G., Sun Z. (2013). Graphene-MnO_2_ nanocomposite modified carbon ionic liquid electrode for the sensitive electrochemical detection of rutin. Sens. Actuat. B Chem..

[22] Cheng H., Liu J., Sun Y., Zhou T., Yang Q., Zhang S., Zhang X., Li G., Sun W. (2020). A fungus-derived biomass porous carbon–MnO_2_ nanocomposite-modified electrode for the voltammetric determination of rutin. RSC Adv..

[23] Parveen N., Ansari S.A., Ansari M.Z., Ansari M.O. (2022). Manganese oxide as an effective electrode material for energy storage: A review. Environ. Chem. Lett..

[24] Nagles E., Hurtado J.J. (2023). Electrochemical Determination of Tartrazine in Foods by Voltammetry with a Screen-printed Carbon/Chitosan-MnO_2_ Microcomposite Electrode. Electroanalysis.

[25] He Q., Liu J., Liu X., Li G., Deng P., Liang J. (2018). Manganese dioxide Nanorods/electrochemically reduced graphene oxide nanocomposites modified electrodes for cost-effective and ultrasensitive detection of Amaranth. Colloids Surf. B Biointerfaces.

[26] Thiagarajan S., Tsai T.H., Chen S.M. (2011). Electrochemical Fabrication of Nano Manganese Oxide Modified Electrode for the Detection of H_2_O_2_. Int. J. Electrochem. Sci..

[27] Gowda J.I., Hanabaratti R.M., Pol P.D., Sheth R.C., Joshi P.P., Nandibewoor S.T. (2022). Manganese oxide nanoparticles modified electrode for electrosensing of antipsychotic drug olanzapine. Chem. Data Collect..

[28] Duan X., Yang J., Gao H., Ma J., Jiao L., Zheng W. (2012). Controllable hydrothermal synthesis of manganese dioxide nanostructures: Shape evolution, growth mechanism and electrochemical properties. CrystEngComm.

[29] Cosco-Salguero G.A., Nagles E., Angulo-Cornejo J., Hurtado-Murillo L.C., Hurtado J.J. (2026). Carbon Paste Electrode Developed from Recycled Manganese Oxide from Batteries and Its Application in the Electrochemical Detection of Paracetamol. ChemistrySelect.

[30] Yang G., Park S.J. (2019). Conventional and microwave hydrothermal synthesis and application of functional materials: A review. Materials.

[31] Pathak Y.A., Mane A.T., More P.D., Thube S.G. (2025). Controlled Hydrothermal Growth of α-MnO_2_ Nanorods: Effect of Temperatureon Electrochemical Performance. ChemistrySelect.

[32] Wei Y., Wang G., Li M., Wang C., Fang B. (2007). Determination of rutin using a CeO_2_ nanoparticle-modified electrode. Microchim. Acta.

[33] Nagles E., Penagos-Llanos J., García-Beltrán O. (2018). Determination of Rutin in Drinks Using an Electrode Modified with Carbon Nanotubes-Prussian Blue. J. Anal. Chem..

[34] Santanatoglia A., Navarini L., Angeloni S., Caprioli G. (2025). Quercetin derivatives in roasted Coffea arabica and its popular beverages. Food Chem..

